# The novel H10N3 avian influenza virus acquired airborne transmission among chickens: an increasing threat to public health

**DOI:** 10.1128/mbio.02363-24

**Published:** 2024-12-16

**Authors:** Xiaoquan Wang, Huiyan Yu, Yahao Ma, Pinghu Zhang, Xiyue Wang, Jianyu Liang, Xiuling Zhang, Ruyi Gao, Xiaolong Lu, Wenhao Yang, Yu Chen, Min Gu, Jiao Hu, Xiaowen Liu, Shunlin Hu, Daxin Peng, Xian Qi, Changjun Bao, Kaituo Liu, Xiufan Liu

**Affiliations:** 1 Joint International Research Laboratory of Agriculture and Agri-Product Safety, The Ministry of Education of China, Yangzhou University, Yangzhou, Jiangsu, China; 2 Key Laboratory of Avian Bioproducts Development, Ministry of Agriculture and Rural Affairs, Yangzhou University, Yangzhou, Jiangsu, China; 3 Jiangsu Key Laboratory of Zoonosis, Yangzhou, Jiangsu, China; 4 Jiangsu Co-innovation Center for Prevention and Control of Important Animal Infectious Diseases and Zoonosis, Yangzhou University, Yangzhou, Jiangsu, China; 5 Jiangsu Provincial Center for Disease Control and Prevention, Nanjing, Jiangsu, China; 6 Yangzhou Center for Disease Control and Prevention, Yangzhou, Jiangsu, China; Virginia Polytechnic Institute and State University, Blacksburg, Virginia, USA; University of South Dakota, Vermillion, South Dakota, USA

**Keywords:** avian influenza virus, novel H10N3, replication, airborne transmission, public health

## Abstract

Following two human infections with the H10N3 avian influenza virus (AIV) in 2021 and 2022, a third case was discovered in Yunnan, China, in 2024, raising concerns about the potential for future pandemics. Recent studies have indicated that novel H10N3 viruses are highly pathogenic in mice and can be transmitted between guinea pigs via respiratory droplets without prior adaptation. However, the biological characteristics of novel H10N3 in poultry have not been fully elucidated. Our findings revealed that H10 subtype AIVs are predominantly prevalent in waterfowl. Notably, H10N8 and H10N3 viruses that have infected humans were primarily isolated from chickens. For the first time, double basic hemagglutinin cleavage sites (motif PEIKQGR↓GL) were identified in novel H10N3 AIVs, which exhibit enhanced replication in chickens, and can be transmitted between chickens through direct contact and respiratory droplets. Animal experimental studies demonstrated that ducks are also susceptible to H10N3 viruses and that the virus is transmissible through direct contact, suggesting a greater risk of transmission and recombination. Serological studies conducted among poultry workers suggest that while the human population was largely naïve to H10N3 infection, sporadic and undetected human infections did occur, indicating a potential increasing trend. These data further emphasize the growing threat to public health posed by zoonotic H10N3 subtype AIVs.

**IMPORTANCE:**

Exposure to poultry in live poultry markets (LPMs) is strongly associated with human infection with avian influenza viruses (AIVs), with chickens being the most common species found in these markets in China. The prevalence of AIVs in chickens, therefore, increases the risk of human infection. Notably, the main host of the novel H10N3 virus has shifted from waterfowl to chickens, and the virus can be transmitted between chickens via respiratory droplets, posing a potential risk of a pandemic within poultry populations. The novel H10N3 virus also remains sensitive to ducks and can be transmitted through direct contact, which means a greater risk of transmission and recombination. Significantly, the human population remains largely naïve to H10N3 infection, but sporadic seropositivity among poultry workers indicates previous exposure to H10 subtype AIVs. Therefore, a comprehensive surveillance of the novel H10N3 viruses in poultry is imperative. Effective control of the virus within poultry populations could significantly reduce the risk of emerging human infections.

## INTRODUCTION

The threat of avian influenza viruses (AIVs) to public health worldwide is alarmingly on the rise (1, 2). H5Ny and H7N9 subtype AIVs have already caused 965 and 1,568 cases of human infection, respectively, manifesting severe clinical symptoms and high fatality rates (3). Moreover, in the past 5 years, numerous subtypes of AIVs, such as H7N4, H10N3, H3N8, and H10N5, have infected humans for the first time (4–8). The potential threat posed by these novel reassortant AIVs on public health remains uncertain.

The H10Nx subtype of AIV continues to remain prevalent globally over an extended period of time (9–15). This subtype is able to rapidly reassort with circulating poultry strains and goes undetected due to its low pathogenicity in chickens. In recent years, the occurrence of H10Ny subtype AIVs infecting humans has become more frequent, including subtypes H10N8 (2013) (16), H10N3 (2021) (5, 17), and H10N5 (2023) (8), which now pose a newly recognized threat to public health security. The first reported human case of H10N3 infection occurred in Jiangsu, China, in 2021 (5). To date, three cases have been documented globally, with the latest case emerging from Yunnan Province in February 2024 (3). Crucially, as H10N3 continues to circulate within poultry populations (18–21), further sporadic cases in humans could potentially be identified in the future. Consequently, the potential public health threat posed by H10 AIVs should not be underestimated.

Our previous studies indicated that human H10N3 originates from poultry, and both human-derived and chicken-derived H10N3 viruses exhibit a high affinity for human-type receptors (20–22). Additionally, several chicken-derived viruses, without prior adaptation, were found to be highly pathogenic in mice and capable of transmission between guinea pigs through respiratory droplets (21, 22). These findings suggest that these viruses may have the potential to evolve into a pandemic threat within the human population.

Human infection with AIVs, including H10N3, is primarily linked to exposure to infected poultry, with no evidence of sustained transmission between humans. Therefore, an H10N3 pandemic among poultry significantly enhances the threat to public health security. In our study, we utilized a human-derived virus (A/Jiangsu/428/2021) and four chicken-derived viruses isolated from eastern China from 2021 to 2022 as model strains to investigate adaptation in poultry. We observed that these chicken-derived viruses were well adapted in chickens, and several strains were capable of transmitting between chickens via respiratory droplets, demonstrating their potential to spread extensively within poultry populations. Moreover, in ducks, these novel H10N3 viruses demonstrated the ability to replicate in individual organs and exhibited contact transmission capabilities. Our research provides critical insights for monitoring novel H10N3 strains with potential pandemic implications.

## RESULTS

### Host tropism and zoonotic infection risk of H10 subtype AIVs

**Fig 1:**
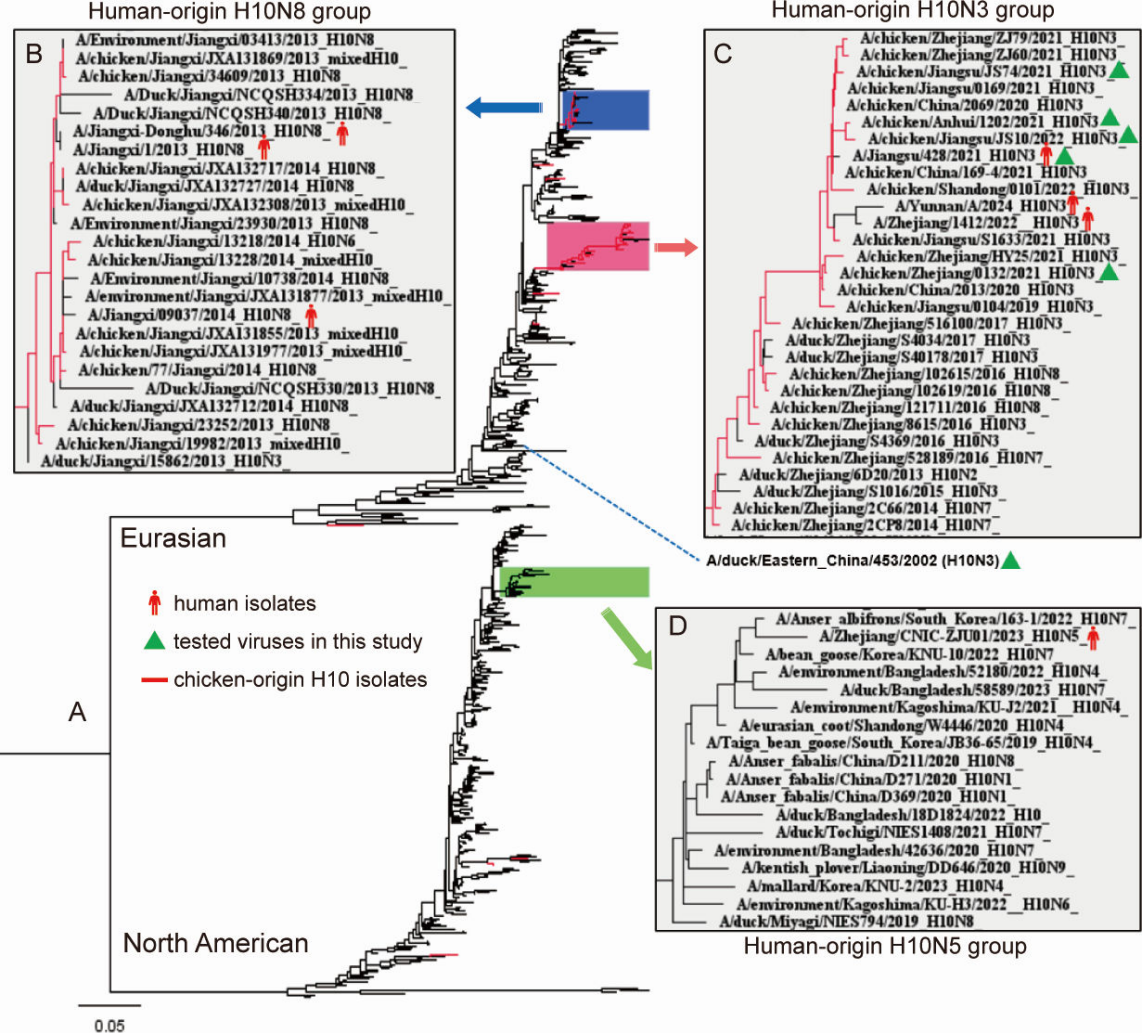
Phylogenetic analysis of hemagglutinin gene of the H10 subtype avian influenza viruses. (**A**) Phylogenetic tree of the hemagglutinin gene of the H10 subtype AIVs. Human-origin (**B**) H10N8, (**C**) H10N3, and (**D**) H10N5 groups. Human-origin H10 viruses are highlighted with red humanoid cartoon logos. H10N3 viruses, which were tested in animal models in this study, are highlighted with green triangles. The branches of chicken-origin H10 viruses are painted in red.

Host range is a critical predictor of the potential risk AIVs pose to humans. To evaluate host preference, we collected the hemagglutinin (HA) sequences of all H10 subtype viruses from the GISAID and NCBI databases, totaling 3,575 sequences. To reduce statistical bias caused by the centralized upload of highly homologous sequences, we down-sampled the data set using BioAider with a 99.5% threshold. This resulted in 612 representative isolates, including three human-derived H10N8 viruses, three human-derived H10N3 viruses, and one human-derived H10N5 virus. As illustrated in Fig. 1A, the human-derived H10N8 and H10N3 isolates clustered within the Eurasian lineage, forming two distinct sub-clades. Additionally, the human-derived H10N5 virus fell within the North American lineage.

**Fig 2:**
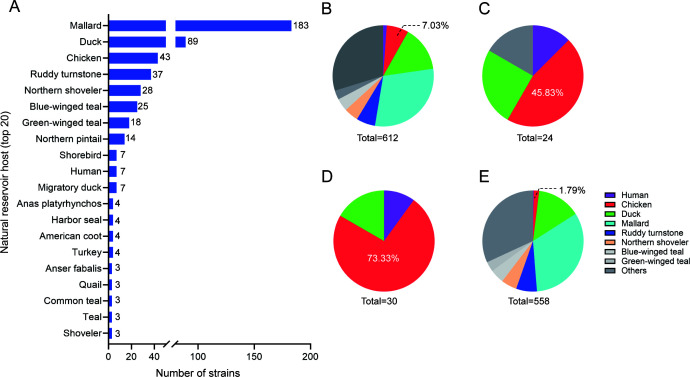
Host distribution of H10 subtype avian influenza viruses. (**A**) The top 20 nature hosts of H10 subtype AIVs. (**B**) Host distribution of H10 subtype AIVs. Host distribution of human-origin (**C**) H10N8 and (**D**) H10N3 group strains. (**E**) Host distribution of no-human-origin H10N8 and H10N3 group strains. All the public data in GenBank and GISAID used in this study were up to date as of 28 May 2024, and representative isolates were selected by down-sampled using BioAider (v1.334) with a 99.5% (cd99.5 data set) threshold.

The H10 subtype AIVs have a broad host range, encompassing wild birds, domestic poultry, mammals, and even humans. As shown in Fig. 2A, the primary natural hosts of H10 subtype AIVs include mallards (183 strains), ducks (89 strains), chickens (43 strains), ruddy turnstones (37 strains), northern shovelers (28 strains), blue-winged teals (25 strains), green-winged teals (18 strains), and northern pintails (14 strains). Notably, with the exception of chickens, all these hosts are wild birds and waterfowl. For H10 subtype AIVs, chicken-derived isolates account for 7.03% (43/612) (Fig. 2B). It is noteworthy that in the human-origin H10N8 and H10N3 groups, chicken-derived isolates account for 45.83% (11/24) (Fig. 2C) and 73.33% (22/30) (Fig. 2D), respectively. In contrast, among strains outside these two groups, chicken-derived isolates account for just 1.79% (10/558) (Fig. 2E). These results suggest that the prevalence of H10 subtype AIVs in chickens may be associated with human infections.

### Replication of novel H10N3 in chickens

**Fig 3:**
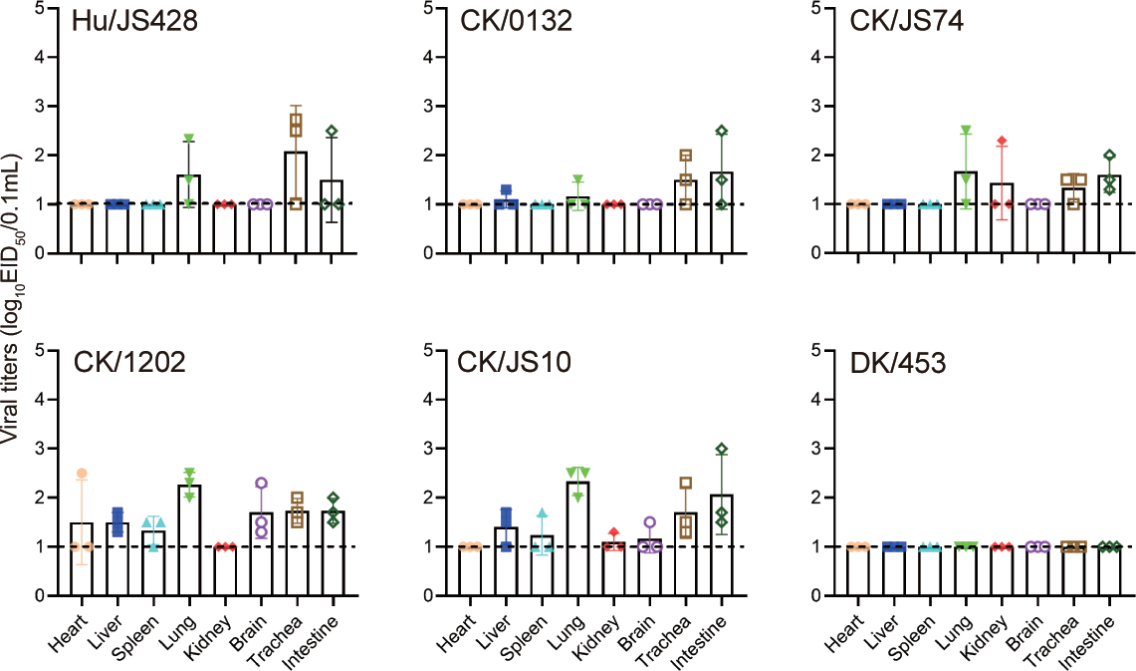
Replication of novel H10N3 viruses in chickens. Six-week-old SPF chickens were challenged with 10^6.0^ EID_50_ of indicated virus, and three chickens were euthanized at 3 dpi. Virus titers in different organs (heart, liver, spleen, lungs, kidney, brain, trachea, and intestine) were determined by EID_50_ assay on chicken eggs. Horizontal dashed lines indicate the lower limit of detection.

Our previous study indicated that the novel H10N3 viruses were low pathogenic in chickens (22). To assess *in vivo* virus replication, the chickens were infected with 10^6.0^ 50% embryo infectious dose (EID_50_) viruses, and the heart, liver, spleen, lungs, kidneys, brain, trachea, and intestine were collected for virus titration at 5 days post-inoculation (dpi) (Fig. 3). Although the human isolate (Hu/JS428) replicated only in individual organs, like lung, trachea, and intestine, the chicken-derived viruses, notably CK/1202 and CK/JS10, replicated in a broader range of organs, although viral titers were lower. Interestingly, no viral replication was detected in any organ after the duck-derived H10N3 strain (DK/453) infected the chickens, and serological results showed no successful infection. Overall, the novel H10N3 viruses, both the human isolate and chicken isolates, could be effectively replicated in chickens, although the replication level was different. However, the duck-derived virus strain isolated in 2002 could not effectively infect chickens.

### Histopathological analysis

**Fig 4:**
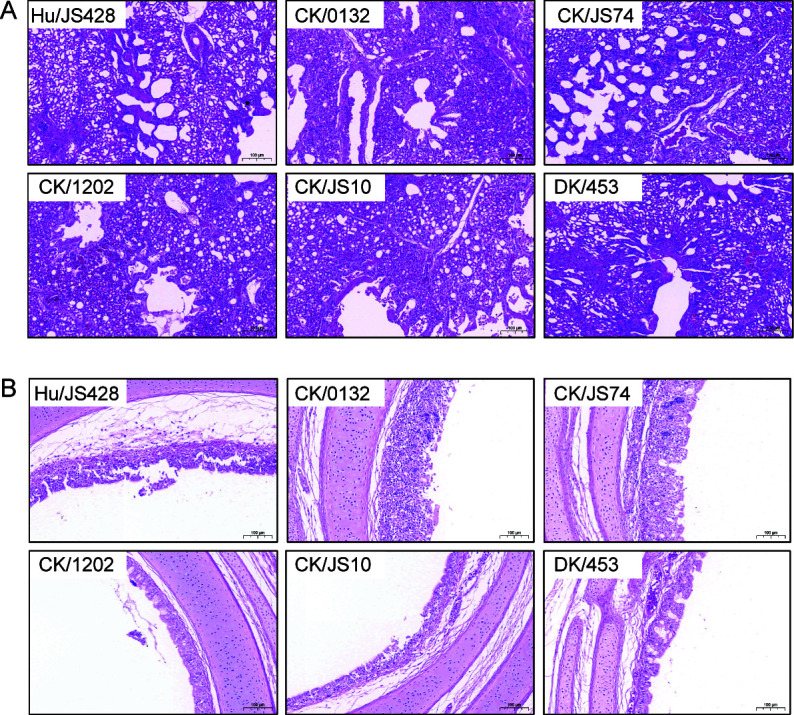
Tissue damage caused by novel H10N3 viruses in chickens. (**A**) Lung and (**B**) trachea lesions caused by novel H10N3 viruses in chickens. Lungs and tracheas collected from chickens intranasally inoculated with 50 µL of the specified viruses at 10^6.0^ EID_50_ were fixed with formalin, embedded in paraffin, and stained with hematoxylin and eosin.

The chickens were euthanized on day 5 after infection with 10^6.0^ EID_50_ of the test virus, and their lung and trachea samples were collected. Hematoxylin and eosin (H&E) staining was performed to investigate the pathological changes. As shown in Fig. 4A, alveolar epithelial cell shedding, a small amount of inflammatory cell infiltration, and some red blood cell exudation could be observed in the alveolar walls and cavity in the lung tissues, despite the degree of pathological changes of different novel H10N3 viruses being not consistent. After novel H10N3 infection, the tracheae exhibited adhesion, lodging, and partial exfoliation of cilia (Fig. 4B). In addition, no significant pathological changes were present in the lungs of DK/453-infected chickens. Overall, our results indicated that the novel H10N3 targets the respiratory tract and causes pathological lesions.

### Transmission of H10N3 in chickens

**Fig 5:**
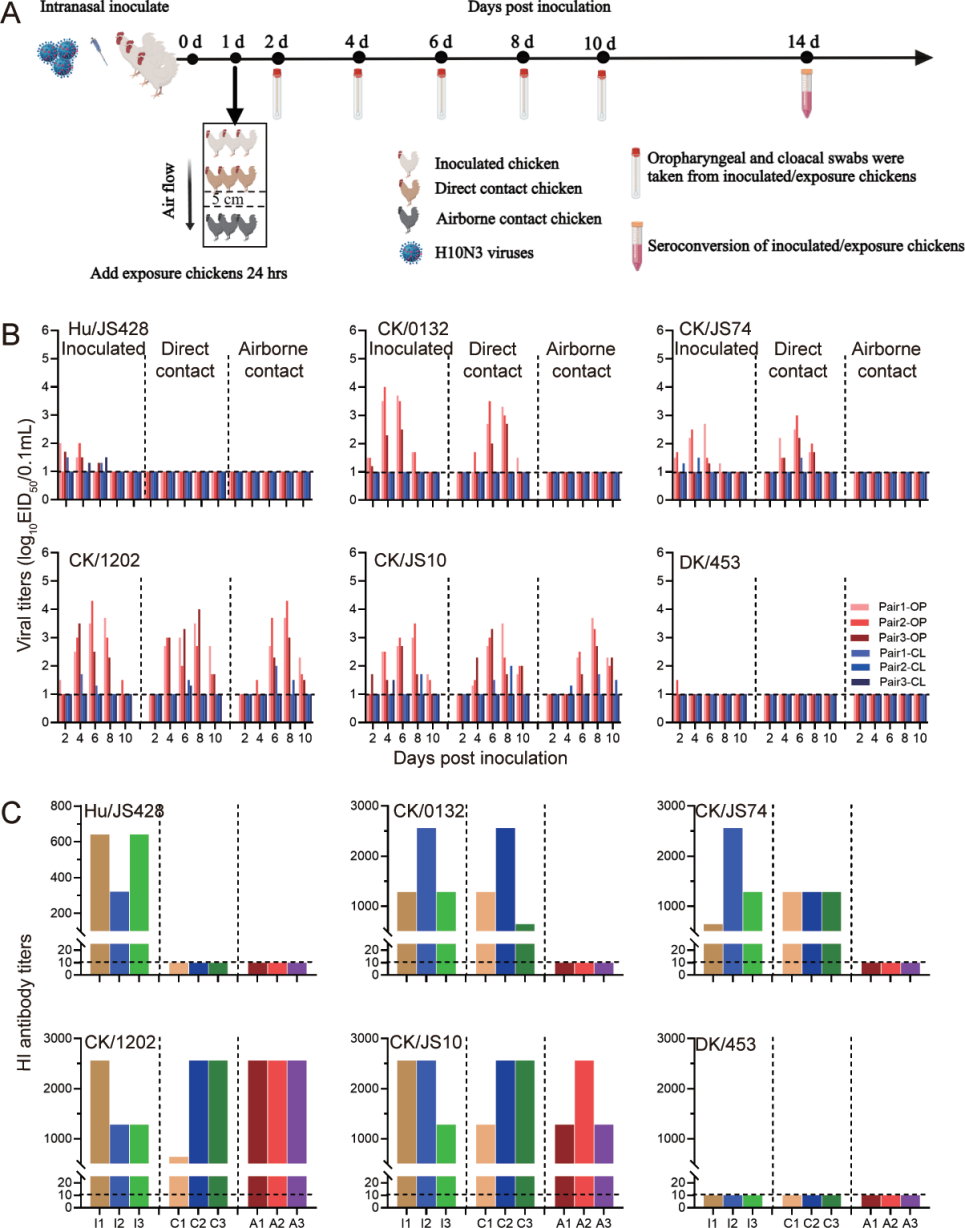
Horizontal transmission of the novel H10N3 viruses in chickens. (**A**) Diagram of the experimental procedure. Briefly, groups of three chickens were inoculated i*.*n. with 10^6.0^ EID_50_ of the indicated viruses: at 24 hpi, three additional chickens were placed into the same isolation units for the direct-contact transmission studies, and another three chickens were housed in adjacent cages (5 cm apart) to monitor airborne transmission. Oropharyngeal (OP) and cloacal (CL) swabs for virus shedding detection were collected every other day from all chickens from 2 days after initial infection. Seroconversion was analyzed by HI assay at 14 dpi. (**B**) Virus shedding of infected and exposed chickens. Virus titers were determined by EID_50_ assay in chicken eggs. Each color bar represents the virus titer from oropharyngeal and cloacal swabs from an individual animal. (**C**) Seroconversion of infected and exposed chickens. I, infected; C, contacted; A, airborne contacted. Horizontal dashed lines indicate the lower limit of detection.

For respiratory viruses, efficient transmissibility often caused pandemics (23). Therefore, we assessed the transmission of each virus from infected chickens to direct-contact chickens, and from infected chickens to airborne-contact chickens. Virus transmission was determined by titration of nasal washes in eggs and testing for seroconversion at 14 dpi. As shown in Fig. 5B, the human-derived Hu/JS428-infected chickens could shed the virus through the throat and cloaca. The chickens began shedding the virus at 2 dpi, and this persisted for 6 days, with a peak virus titer <10^2.0^ EID50/mL. All the direct-contact and airborne-contact chickens did not detected virus shedding and seroconverted (Fig. 5). Chicken-derived H10N3 viruses were also detected in all infected chickens, and the chickens began shedding virus at 2 dpi and this persisted for 10 days, with a peak virus titer <10^4.3^ EID_50_/mL (Fig. 5B). All direct-contact chickens exhibited virus shedding and had seroconverted by 14 dpi, with hemagglutinin inhibition (HI) titers >1,280 (Fig. 5), indicating that chicken-derived viruses can be transmitted to other chickens through direct contact with infected one. Notably, with the chicken-derived isolate CK/1202 and CK/JS10, virus shedding and seroconversion were detectable in all airborne-contact chickens (Fig. 5), which implies potential transmission through respiratory droplets. In addition, early isolate, duck-derived H10N3 virus (DK/453) was detected in only one infected-chicken throat sample at 2 dpi, with a virus titer of 10^1.5^ EID_50_/mL, and no virus shedding and seroconversion were detectable in all infected, direct- and airborne-contact chickens (Fig. 5). These results indicate that some chicken-derived novel H10N3 viruses have acquired airborne transmission capabilities during replication and evolution in chickens.

### Pathogenicity and replication of novel H10N3 in ducks

**Fig 6:**
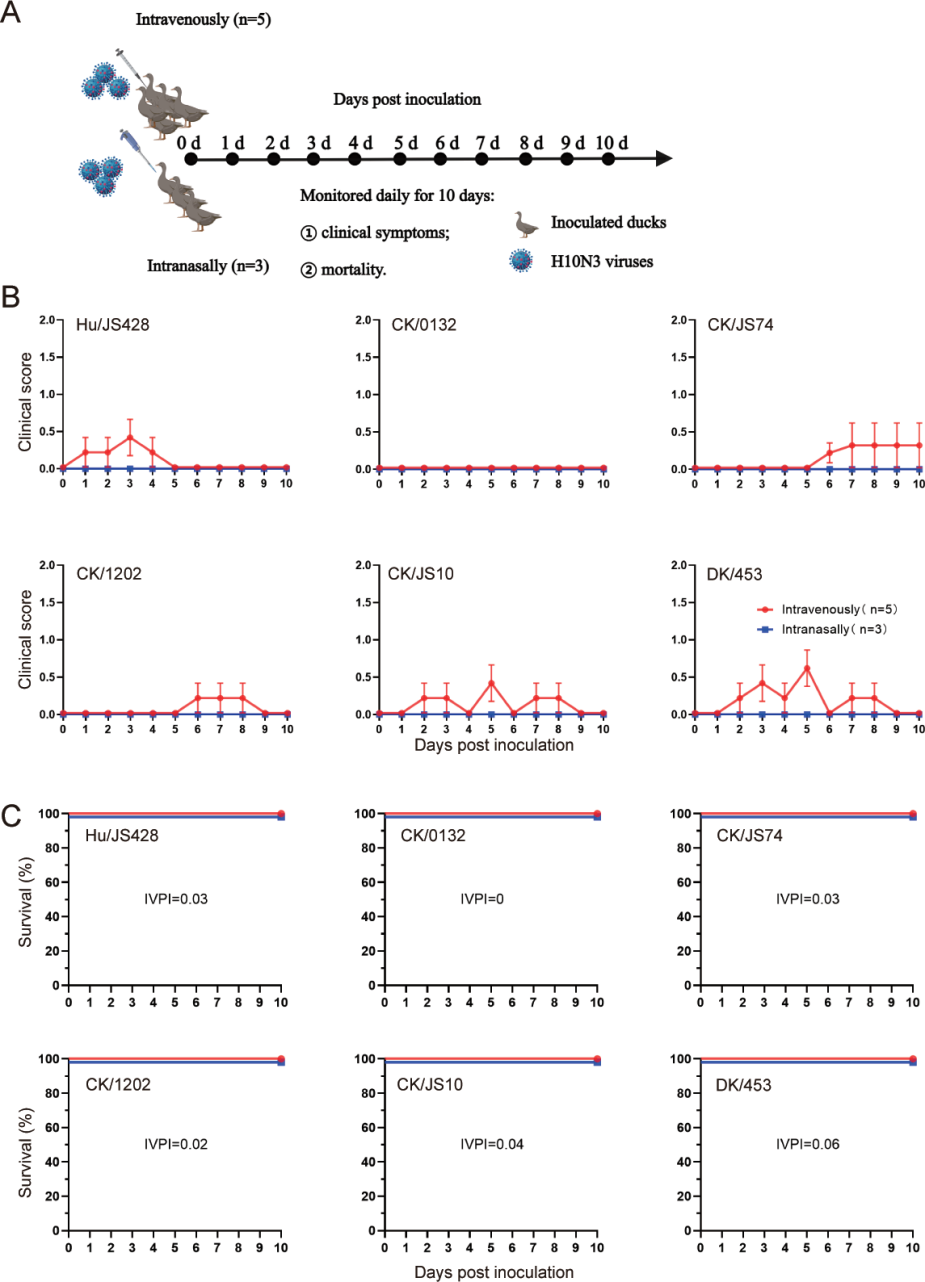
The pathogenicity of novel H10N3 in ducks. (**A**) Diagram of the experimental procedure. First, 3-week-old, non-immune mallard ducks (*n* = 5 per group) were inoculated intravenously with 100 µL of a 1:10 dilution of infectious allantoic fluid in PBS to determine the IVPIs. Second, 3-week-old, non-immune mallard ducks (*n* = 3 per group) were inoculated i*.*n. with 10^6.0^ EID_50_ of the indicated viruses in a volume of 100 µL. Clinical symptoms and survival were monitored daily for 10 dpi. (**B**) Clinical symptoms of infected ducks. The clinical symptoms were scored as follows: 0, at healthy state; 1, signs of spirit, inactivity, and loss of appetite; 2, as above and little mobility or paralysis; 3, death. (**C**) Survival of infected ducks.

Wild aquatic birds, including ducks, are the natural reservoirs for low-pathogenic avian influenza viruses (LPAIVs) (24, 25). However, few studies have focused on experimental H10N3 virus infections in ducks. Consequently, this study investigated the virulence of H10N3 viruses in ducks using both intravenous and intranasal inoculations (Fig. 6). The intravenous pathogenicity index (IVPI) scores for the ducks were below 0.06, although individual ducks exhibited mild clinical symptoms that subsided after several days, indicating that the novel H10N3 viruses were of low pathogenicity. In addition, when infected intranasally with 10^6.0^ EID_50_ of each H10N3 virus and monitored daily for 10 days, all ducks showed no clinical symptoms and all survived the infection. Overall, both the human isolate and the chicken isolates in this study demonstrated low pathogenicity to ducks.

**Fig 7:**
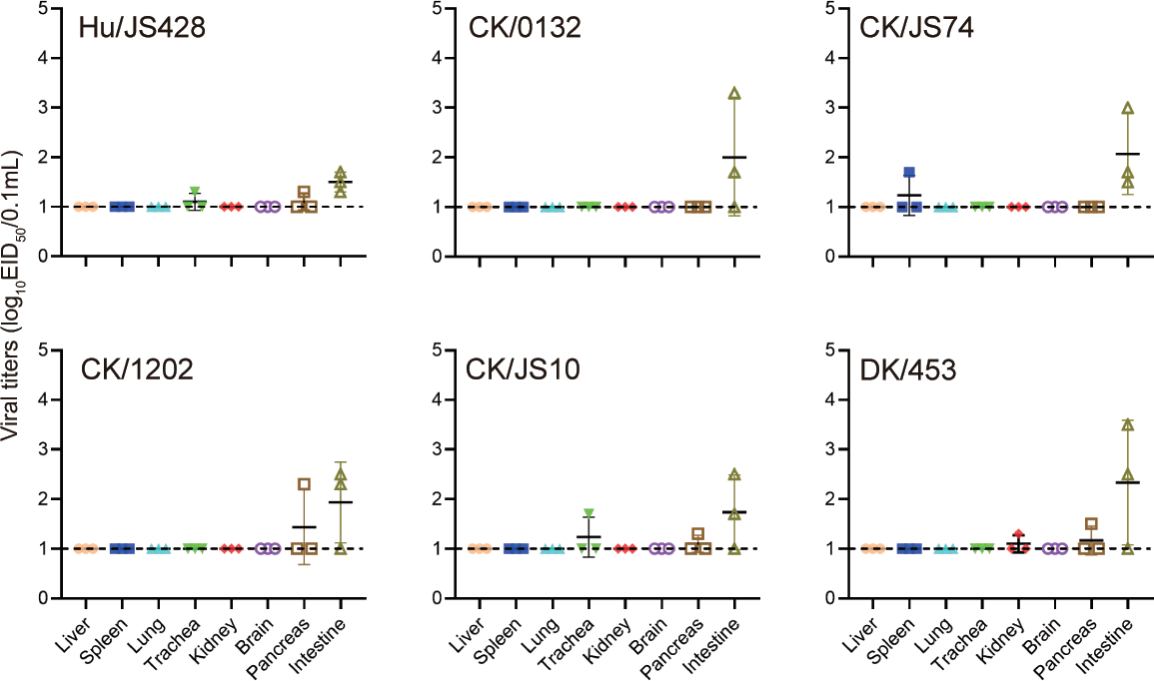
Replication of novel H10N3 viruses in ducks. Three-week-old, non-immune mallard ducks were challenged with 10^6.0^ EID_50_ of indicated virus, and three ducks were euthanized at 3 dpi. Virus titers in different organs (liver, spleen, lungs, trachea, kidney, brain, pancreas, and intestine) were determined by EID_50_ assay on chicken eggs. Horizontal dashed lines indicate the lower limit of detection.

To assess *in vivo* virus replication, the ducks were i*.*n. infected with 10^6.0^ EID_50_ viruses, and the liver, spleen, lung, kidney, brain, pancreas, and intestine were collected for virus titration at 3 dpi (Fig. 7). The human-derived virus (Hu/JS428) can replicate in the trachea, pancreas, and intestine of the ducks, and the replication of the virus was maintained at a relatively low level (1.3 to 1.7 log_10_ EID_50_/0.1 mL). The chicken-derived viruses (including CK/0132, CK/JS74, CK/1202, and CK/JS10) also only replicated in individual organs, like spleen, trachea, pancreas, and intestine, and exhibited efficient replication ability in the intestine, with a peak virus titer of 10^3.3^ EID_50_/0.1 mL. The duck-derived virus can replicate in the kidney, pancreas, and intestine of the ducks, although the replication of the virus was maintained at a relatively low level (1.3 to 3.5 log_10_ EID_50_/0.1 mL). These experimental infection studies indicate that the novel H10N3 virus can asymptomatically infect and replicate in the intestine of ducks.

### Transmission of novel H10N3 in ducks

**Fig 8:**
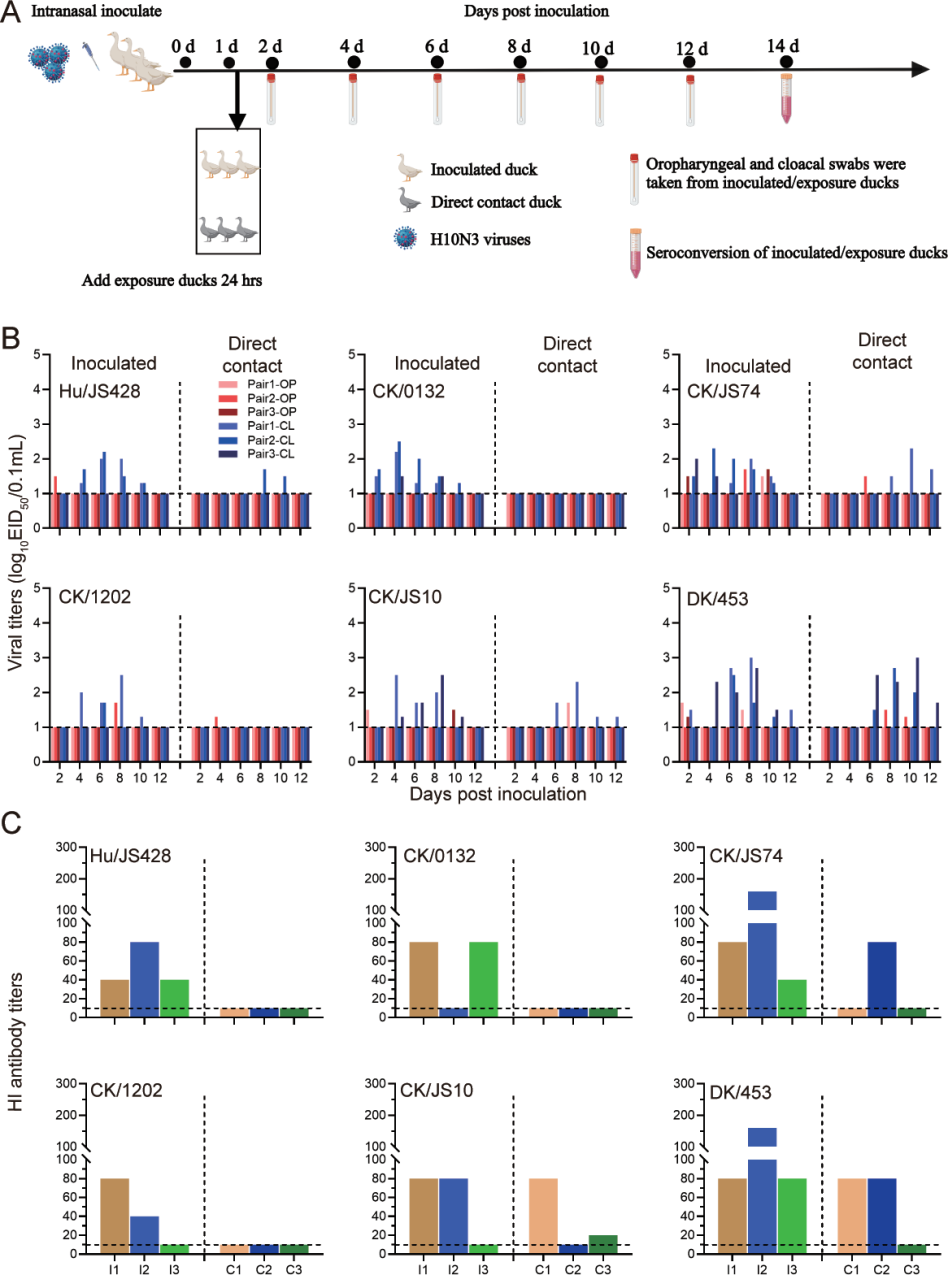
Horizontal transmission of the novel H10N3 viruses in ducks. (**A**) Diagram of the experimental procedure. Briefly, groups of three ducks were inoculated i*.*n. with 10^6.0^ EID_50_ of the indicated viruses: at 24 hpi, three additional ducks were placed into the same isolation units for the direct-contact transmission studies. Oropharyngeal (OP) and cloacal (CL) swabs for virus shedding detection were collected every other day from all ducks from 2 days after initial infection. Seroconversion was analyzed by HI assay at 14 dpi. (**B**) Virus shedding of infected and exposed ducks. Virus titers were determined by EID_50_ assay in chicken eggs. Each color bar represents the virus titer from oropharyngeal and cloacal swabs from an individual animal. (**C**) Seroconversion of infected and exposed ducks. I, infected; C, contacted. Horizontal dashed lines indicate the lower limit of detection.

To evaluate the risk of the novel H10N3 virus spreading among ducks, we examined the direct transmission ability of these H10N3 in ducks (Fig. 8). In this study, virus transmission was determined by titration of oropharyngeal and cloacal swabs in eggs and testing for seroconversion at 14 dpi. The novel H10N3, including human-derived and chicken-derived viruses, can all be detected in the inoculated ducks, and this persisted for 10 dpi, with a peak virus titer of 10^2.5^ EID_50_/0.1 mL. As shown in Fig. 8C, seroconversion of the Hu/JS428, CK/0132, CK/JS74, CK/1202, and CK/JS10 viruses was observed in 3/3, 2/3, 3/3, 2/3, and 2/3 inoculated animals. Nevertheless, their transmissibility was variable. According to the combined viral shedding and seroconversion result, only CK/JS74 and CK/JS10 can transmit viruses from inoculated ducks to direct-contact ducks, although the transmission efficiency is relatively low. In addition, the early isolate, duck-derived H10N3 virus (DK/453) can be detected in the inoculated ducks, and this persisted for 12 dpi, with a peak virus titer of 10^3.0^ EID_50_/0.1 mL. Notably, DK/453 also can be detected in the direct-contact ducks, with a peak virus titer of 10^3.0^ EID_50_/0.1 mL, suggesting that it could be transmitted to ducks by direct contact. The serological results showed that 3/3 inoculated and 2/3 direct-contact ducks had a successful infection. Overall, these results indicate that the novel H10N3 viruses pose a high risk of infection to ducks.

### Serological results from poultry workers

Exposure to poultry is strongly associated with human infection with AIVs, and antibodies are the most efficient way the immune system fights viruses. To assess the level of pre-existing immunity in humans and the proportion of people who are or have been infected but have not been detected, we conducted a serological surveillance for antibodies to H10 subtype AIVs among poultry workers in Jiangsu Province. From 2023 to 2024, we collected a total of 2,769 serum samples from poultry workers. The human-derived H10N3 virus strain Hu/JS428 was used as the antigen for this study. As shown in Table 1, we collected 637 and 943 serum samples from poultry workers in 2021 and 2022, respectively, and there is one positive sample in each of the two years. It is noteworthy that among the 790 serum samples collected in 2023, four were found to be positive, while among the 399 serum samples collected in 2024, six were identified as positive, resulting in a positivity rate of 1.5%. These findings suggest that the human population was largely naïve to H10N3 infection during the study period. However, sporadic and undetected human infections did occur, indicating a potential increasing trend.

**TABLE 1:** Seropositive rate of H10 subtype AIVs in poultry workers in Jiangsu Province

Antigen	2021 (*n* = 637)		2022 (*n* = 943)		2023 (*n* = 790)		2024 (*n* = 399)	
	No. positive	% positive	No. positive	% positive	No. positive	% positive	No. positive	% positive
A/Jiangsu/428/2021	1	0.16	1	0.11	4	0.51	6	1.5

## DISCUSSION

Wild animals are natural reservoir hosts for a variety of pathogen, and for many zoonoses, livestock serve as an epidemiological bridge between wildlife and human infections (26, 27). Human infection with AIVs is primarily linked to exposure to infected poultry (28, 29); therefore, the widespread prevalence of AIVs in poultry has increased the threat to public health safety. This study aimed to investigate the epidemic potential of the novel H10N3 virus with zoonotic ability in poultry.

For H10 subtype AIVs, wild aquatic birds are natural reservoirs (9), and our statistical results are also in line with this point of view (Fig. 2). Based on the HA phylogeny and host tropism analysis, we found that the human-derived H10N8 and H10N3 viruses were all clustered with chicken-derived isolates. The same phenomenon has been found in other subtypes of AIVs, such as H9N2, H5Ny, and H7N9, which were or have been widely prevalent in China and have caused a number of human infections (30–32). The spread of AIVs in chickens means there are more opportunities for contact with humans, which provides more favorable conditions for infecting humans. In addition, the first human case of H10N5 (in combination with seasonal influenza A H3N2) was confirmed in China in 2023 and unfortunately succumbed to the infection (8). The results from our research show that the human-derived H10N5 HA gene may be derived from the H10N7 virus prevalent in Korean waterfowl but not yet widely prevalent among chickens. This phenomenon was not consistent with other subtype AIVs. One possible reason is that there has only been one reported case of human infection with H10N5, and the epidemiology of this strain is still largely unexplored. The scant information highlights the necessity for ongoing monitoring and research to more comprehensively understand the potential risks and characteristics of H10N5.

The primary host of novel H10N3 virus has transformed from wild birds and waterfowl to chickens, but the biological characteristics in chickens remain relatively unexplored. Our previous study indicated that the novel H10N3 viruses had low pathogenicity in chickens (22), so it could spread quietly among poultry without being detected. In this study, we discovered that 2/4 chicken-derived H10N3 viruses (CK/1202 and CK/JS10) have become capable of transmission through respiratory droplets in chickens. We found these two viruses exhibited two basic amino acid insertions at the HA cleavage sites through the genome analysis. This phenomenon is exceedingly rare in H10N3 subtype AIVs (Fig. S1). The cleavage activation of HA by host protease is a pivotal step in the influenza virus life cycle and ultimately influences virulence, transmissibility, and adaption to new species (33, 34). The insertion of multiple basic amino acids at the HA cleavage sites has resulted in the transition of H7N9 from low to high pathogenicity (33, 35), and Wen et al. first identified that the novel H3N8 had tri-basic HA cleavage sites (36). Although our previous studies have shown that the novel H10N3 virus is still of low pathogenicity to chickens (22), we also need to exercise increased vigilance toward this phenomenon. Whether the insertion of basic amino acids plays a significant role in airborne transmission remains unclear and is a prime focus of our ongoing investigation. Notably, transmissible respiratory droplets excreted by chickens will facilitate rapid spread in poultry and will increase the risk of infection for workers in farms and live poultry markets (LPMs), directly threatening public health safety. Serological tests among poultry workers were conducted in this study, and results show 12 poultry workers were seropositive to H10N3 from 2021, resulting in a positivity rate of 1.5% (6/399) in 2024. These positive cases indicate that poultry workers had previous exposure to the H10 subtype AIVs, although it may not be H10N3. These data further emphasize the occupational risk posed by zoonotic AIVs.

Wild waterfowl are considered natural reservoirs for AIVs and can carry the virus without showing significant signs of illness, allowing the virus to persist in these populations (37, 38). In addition, waterfowl can shed AIVs to lakes, rivers, and wetlands, where waterfowl are commonly found, which can support the persistence of AIVs due to favorable conditions for virus survival (39). Importantly, the presence of diverse AIV strains in waterfowl populations provides opportunities for viral reassortment, which can lead to the emergence of new strains with potentially higher pathogenicity or transmissibility (38). In this study, we found that the novel H10N3 viruses can replicate in multiple organs of ducks, with relatively high virus titers in the intestine. We also found that infected ducks can continue to excrete the virus through cloaca, resulting in infection in the direct-contact group of ducks (2/5). However, these novel H10N3 viruses did not induce the production of high titers of HI antibody in ducks. The experimental studies revealed that ducks were susceptible to these viruses, which means a greater risk of transmission and recombination of the novel H10N3.

In summary, the H10N3 viruses’ main host shifted from waterfowl to terrestrial poultry, and acquired the ability of airborne transmission during nature adaptation in chickens. Additionally, it also maintained sensitivity to waterfowl. The human population was also naïve to H10N3 infection, but sporadic seropositivity among poultry workers does exist. Thus, a comprehensive surveillance of the novel H10N3 viruses in chickens and wild waterfowl is imperative, and control of the virus endemic in poultry could decrease the risk of emerging human infections.

## MATERIALS AND METHODS

### Viruses

Human H10N3 virus A/Jiangsu/428/2021 (Hu/JS428) was kindly provided by the Jiangsu Provincial Center for Disease Control and Prevention. A/chicken/Zhejiang/0132/2021 (CK/0132), A/chicken/Jiangsu/JS74/2021 (CK/JS74), A/chicken/Anhui/1202/2021 (CK/1202), and A/chicken/Jiangsu/JS10/2022 (CK/JS10) were identified from the tracheal samples of chickens in eastern China from 2020 to 2022 (22). A/duck/Eastern China/453/2002 (DK/453) was isolated from ducks in eastern China in 2002. All viruses were grown and amplified in 9-day-old embryonated chicken eggs and were stored at −80°C for future use.

### Phylogenetic analysis

The genome sequences of H10N3 viruses isolated by our laboratory in eastern China were deposited in GISAID databases (https://www.gisaid.org). The published HA gene sequences of H10Ny were downloaded from the online databases (updated to 28 May 2024) of the NCBI Influenza Research Database (www.ncbi.nlm.nih.gov/genomes/FLU/) and GISAID, respectively. Sequences were aligned using MAFFT (v7.3) and were manually trimmed in MEGA X to obtain the open reading frames, and redundant sequences with the same ID were removed. In order to eliminate statistical bias caused by centralized uploading of highly homologous sequences, representative isolates were selected by down-sampling using BioAider (v1.334) with a 99.5% (cd99.5 data set) threshold. The best-fit nucleotide substitution model was selected using IQ-tree (v1.6.12). The phylogenetic tree of HA was visualized and embellished by FigTree (v1.4.4).

### Chicken challenge experiments

Six-week-old, specific-pathogen-free (SPF) chickens (Vital River Laboratories, Beijing, China) were used to investigate the transmissibility of these novel H10N3 isolates. Groups of three 4-week-old SPF chickens were inoculated i*.*n. with 10^6.0^ EID_50_ of the desired viruses in a volume of 100 µL to investigate the transmissibility of the H10N3 viruses in poultry. At 24 hours post-infection (hpi), three additional naive chickens were placed into the same isolation units for the direct-contact transmission studies, and another three naive chickens per group were housed in adjacent cages (5 cm apart) to monitor airborne transmission. The airflow rates were adjusted from >0.1 to approximately <0.3 m/s, directing airflow from the infected group to the exposed group. The temperature and humidity were set at 22°C and 30 to 40%. To monitor virus shedding, oropharyngeal and cloacal swabs were collected from all chickens every other day for 10 days. Sera were collected from both inoculated and contacted chickens at 14 dpi. Seroconversion was analyzed by hemagglutinin inhibitio (HI) assay.

Additionally, groups of three 4-week-old, SPF chickens were inoculated i*.*n. with 10^6.0^ EID_50_ of the desired viruses in a volume of 100 µL to investigate the replication of the H10N3 viruses in chickens. At 5 dpi, these chickens were euthanized, and the heart, liver, spleen, lungs, kidney, brain, trachea, and intestine were collected aseptically for virus titration. Nine-day-old embryonated SPF chicken eggs were used in the virus titration. The lung and trachea of chickens infected with the indicated viruses were collected and fixed in 10% neutral-buffered formalin, embedded in paraffin, and cut into 4-µm sections. The sections were stained with H&E.

### Duck challenge experiments

Three-week-old, non-immune mallard ducks (*n* = 5 per group) (Yangzhou Experimental Animal Center, Jiangsu, China) were intravenously inoculated with 0.1 mL of a 1:10 dilution in phosphate-buffered saline (PBS) of infectious allantoic fluid to determine the corresponding IVPI, as described by the World Organization for Animal Health (WOAH) (40).

Three-week-old, non-immune mallard ducks were used to investigate the pathogenicity and transmissibility of the novel H10N3 isolates. Groups of six ducks were intranasally challenged with 10^6.0^ EID_50_ of the desired viruses in a volume of 100 µL. At 24 hpi, three additional naive non-immune mallard ducks were placed into the same isolation units for the direct-contact transmission studies. The temperature and humidity were set at 22°C and 30 to 40%. To monitor virus shedding, oropharyngeal and cloacal swabs were collected from all ducks every other day for 12 days and were titrated for viruses on 9-day-old embryonated SPF chicken eggs. Sera were collected from all ducks at 14 dpi; seroconversion was analyzed by HI assay. Additionally, three ducks in the inoculated group were euthanized, and the liver, spleen, lungs, trachea, kidney, brain, pancreas, and intestine were collected aseptically at 3 dpi for viral titration. Nine-day-old embryonated SPF chicken eggs were used in the virus titration.

### Serological survey of the H10N3 virus infection in humans

To determine the risk factor of humans for H10N3 AIV infection, a serological surveillance among live poultry workers was carried out using HI assays in Jiangsu, China, between 2021 and 2024. Human H10N3 virus Hu/JS428 were used as antigens. Standard HI assays were performed using 1% turkey erythrocytes in accordance with WHO guidelines with established procedures. Before the antibody response assay, all serum samples were treated with receptor-destroying enzyme (Denka Seiken) at 37°C for 16 hours and were heat inactivated at 56°C for 30 minutes. HI titer ≥10 was considered positive.

## Data Availability

Genome sequences obtained in this study were submitted to the NCBI database, under the accession numbers: OM802511-OM802519 for HU/JS428, PQ660942-PQ660949 for CK/0132, PQ661122-PQ661129 for CK/JS10, PQ661130-PQ661137 for CK/1202, PQ661138-PQ661145 for CK/JS74, PQ661146-PQ661147 for DK/453. Other relevant data are within the paper.

## SUPPLEMENTAL MATERIAL

Fig. S1 mbio.02363-24-s0001.docx Hemagglutinin cleavage site frequency of H10 subtype AIVs.

ASM does not own the copyrights to Supplemental Material that may be linked to, or accessed through, an article. The authors have granted ASM a non-exclusive, world-wide license to publish the Supplemental Material files. Please contact the corresponding author directly for reuse.
