# Supplementary material for: The novel H10N3 avian influenza virus acquired airborne transmission among chickens: an increasing threat to public health
Source: mBio. 2024 Dec 16;16(2):e02363-24. doi: 10.1128/mbio.02363-24 (PMC11796378; doi:10.1128/mbio.02363-24)
Supplement: Fig. S1 — Hemagglutinin cleavage site frequency of H10 subtype AIVs. [file mbio.02363-24-s0001.docx]

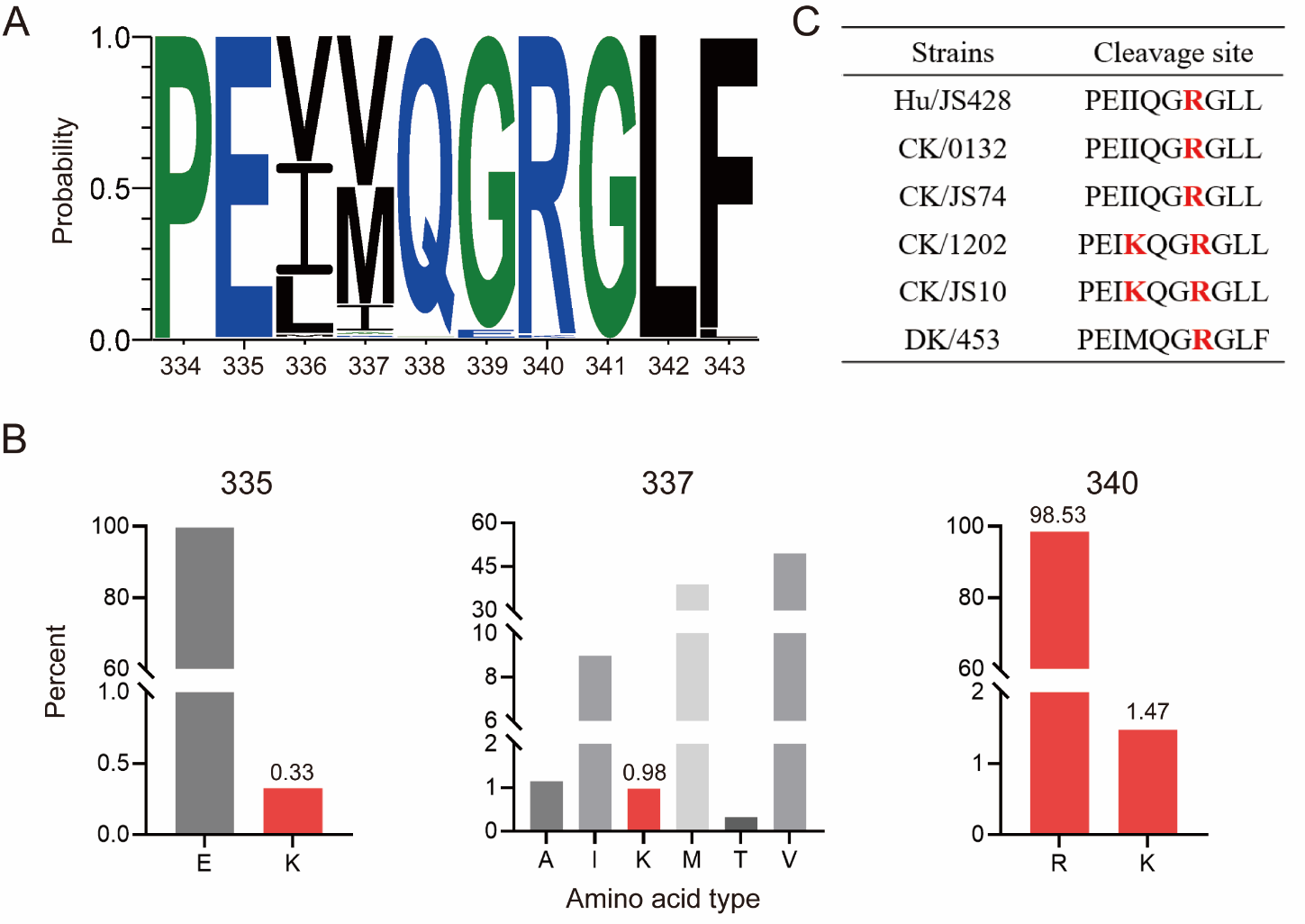
Figure S1 Hemaglutinin cleavage sites frequency of H10 subtype AIVs. All the public data in GenBank and GISAID used in this study were up to date as of May 28, 2024, and representative isolates were selected by down-sampled using BioAider (v1.334) with a 99.5% (cd99.5 dataset) threshold. (A), Frequencies of hemaglutinin cleavage sites were illustrated using Weblogo 3.4 (<http://weblogo.threeplusone.com/>). (B), Frequencies of each position, including 335, 337 and 340 (H10 numbering) in hemaglutinin cleavage sites. (C), Types of hemaglutinin cleavage sites of the strains used in this study.
